# Managing Waldenström’s macroglobulinemia with BTK inhibitors

**DOI:** 10.1038/s41375-022-01732-9

**Published:** 2022-11-19

**Authors:** Christian Buske, Wojciech Jurczak, Joe-Elie Salem, Meletios A. Dimopoulos

**Affiliations:** 1 Institute of Experimental Cancer Research, Comprehensive Cancer Center, University Hospital of Ulm, Ulm, Germany; 2 Department of Clinical Oncology, Maria Skłodowska-Curie National Research Institute of Oncology, Kraków, Poland; 3 Sorbonne University, AP-HP, INSERM CIC-1901, Paris, France; 4 Department of Clinical Therapeutics, National and Kapodistrian University of Athens, Athens, Greece

**Keywords:** B-cell lymphoma, Targeted therapies

## Abstract

Bruton’s tyrosine kinase (BTK) inhibition is one of the treatment standards for patients with relapsed/refractory Waldenström’s macroglobulinemia (WM) and for patients with WM who are unsuitable for immunochemotherapy (ICT). It offers deep and durable responses with a manageable safety profile that is generally favorable compared with ICT regimens. However, the limitations of the first approved BTK inhibitor (BTKi), ibrutinib, include reduced efficacy in patients lacking the characteristic WM mutation (*MYD88*^L265P^) and toxicities related to off-target activity. The risk of atrial fibrillation (AF) and other cardiovascular side effects are a notable feature of ibrutinib therapy. Several next-generation covalent BTKis with greater selectivity for BTK are at various stages of development. In November 2021, zanubrutinib became the first of these agents to be approved by the European Medicines Agency for the treatment of WM. Head-to-head trial data indicate that it has comparable efficacy to ibrutinib for patients with WM overall, although it may be more effective in patients with *CXCR4* mutations or wild-type *MYD88*. In the clinical trial setting, its greater selectivity translates into a reduced risk of cardiovascular side effects, including AF. Acalabrutinib, which is pre-approval in WM, appears to offer similar advantages over ibrutinib in terms of its safety profile. Beyond the next-generation covalent BTKis, non-covalent BTKis are an emerging class with the potential to provide a therapeutic option for patients who relapse on covalent BTKis. In the future, BTKis may be increasingly utilized within combination regimens. Several ongoing trials in WM are investigating the potential for BTKi use in combination with established and novel targeted agents.

## Introduction

Waldenström’s macroglobulinemia (WM) is a rare indolent B-cell lymphoma characterized primarily by bone marrow infiltration by lymphoplasmacytic cells and IgM monoclonal gammopathy [1]. It comprises approximately 1–2% of all cases of non-Hodgkin’s lymphoma in Europe and the USA, with an incidence of ~4 cases per 1 million person-years [2–6]. Prevalence is higher in males than in females and in Caucasians versus other races, and increases with age, with most WM patients aged ≥65 years at the time of diagnosis [4, 5, 7]. It is incurable, with a median survival of 10–12 years [8], and many patients die from causes unrelated to the disease [9].

**Fig. 1:**
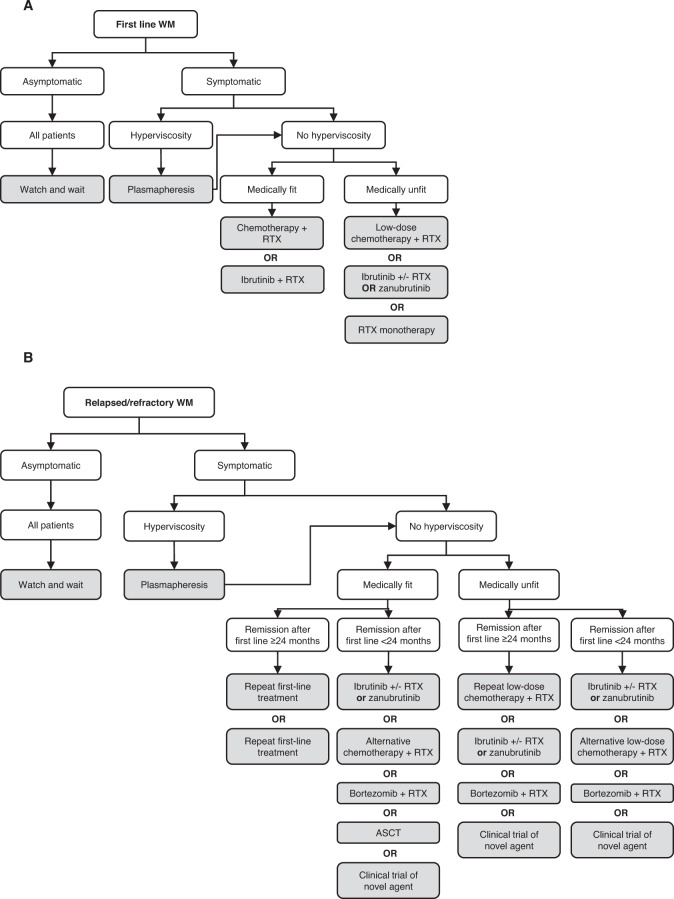
Treatment algorithms for WM. Figure adapted from DGHO-onkopedia guidelines [12]. RTX rituximab.

The emergence of more effective treatments over the last two decades has resulted in improved outcomes for patients [10]. Rituximab-based immunochemotherapy (ICT) regimens were the mainstay of treatment for first-line and relapsed/refractory (R/R) WM [11], but Bruton’s tyrosine kinase inhibitors (BTKi), such as ibrutinib, are now standard for R/R disease (Fig. 1) [12]. Ibrutinib offers deep and durable responses with an acceptable toxicity profile. However, it has limitations, including potentially serious side effects attributed to significant off-target activity [13]. Next-generation BTKis have been developed with the aim of achieving greater target occupancy and selectivity, and this review will provide a practical overview of these agents. The greatest differentiation between the BTKis may be in their safety profiles, and this article will outline the most common and problematic side effects, including ibrutinib-associated AF. We will also consider future developments in the treatment of WM that may overcome some of the limitations of BTKi monotherapy.

## BTK and WM biology

**Fig. 2:**
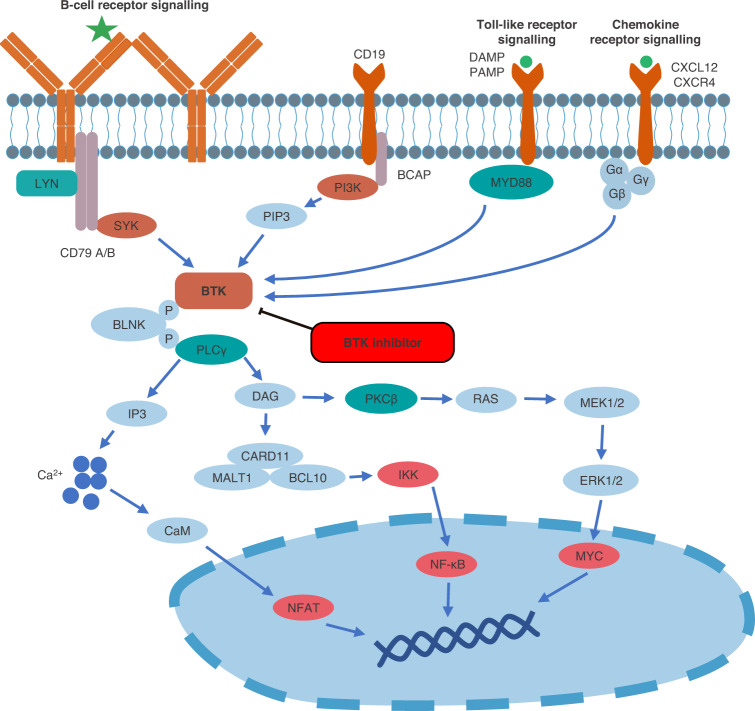
BTK signaling pathways in B cells. BTK has a crucial role in signaling pathways that regulate various aspects of B-cell behavior, including survival and proliferation. Adapted from [96].

BTK belongs to the TEC family of non-receptor kinases, which comprises five kinases (BMX, BTK, ITK, TEC, and TXK), which all have important roles in immunity [14]. BTK, ITK, and TXK appear to be selectively expressed in hematopoietic cells, whereas BMX and TEC are expressed more widely. TEC, for example, is best characterized for its role in T cell development, but is also expressed in the liver, heart, kidney, and ovary [15]. BTK is expressed in B cells and other hematopoietic cell types, including T cells and mast cells [14–17]. Its role is best characterized in B cells, where it acts downstream of the B-cell receptor and within multiple other pathways that are essential for maturation, proliferation, and survival (Fig. 2) [14]. The importance of BTK to B-cell function is highlighted by the severe immunodeficiency disorder X-linked agammaglobulinemia, which is characterized by a lack of mature B cells in the periphery and greatly decreased or absent immunoglobulins. The disease is caused by loss-of-function mutations in *BTK* [18, 19] and provides the rationale for targeting BTK in B-cell malignancies. Of note, besides the described loss-of-function mutations in the *BTK* gene, neither inherited, nor somatic BTK driver mutations are known.

Pertinent to the biology of WM, BTK is involved in toll-like receptor signaling, which also involves the adaptor protein MYD88. The activating *MYD88*^L265P^ mutation, which is present in >90% of patients with WM, causes MYD88 to spontaneously assemble protein complexes that trigger pro-survival signaling along multiple pathways [20]. Activating mutations in the chemokine receptor gene *CXCR4* are also common in patients with WM (~40%) and almost always occur with *MYD88* mutations [20]. CXCR4 is a ubiquitously expressed G protein-coupled receptor that acts as a conventional chemokine receptor. Its natural ligand is CXCL12, and CXCL12-CXCR4 binding leads to activation of an array of signaling pathways involved in cell proliferation and migration. *CXCR4* gene mutations in patients with WM impair CXCR4 desensitization and internalization, which results in prolonged signaling upon binding of CXCL12. This leads to enhanced AKT and subsequent MAPK1/2 signaling, which results in sustained survival signals [21].

Mutational analysis of *MYD88* from bone marrow samples is standard in WM diagnosis. Screening is usually performed initially by allele-specific or reverse transcription PCR for the hallmark L265P mutation with Sanger sequencing used to check for other *MYD88* mutations when L265P is not present [22]. *CXCR4* is not analyzed routinely within clinical practice because of its weaker diagnostic value and the challenges associated with a complex mutational landscape and subclonality [20]. When *CXCR4* analysis is performed, Sanger or next-generation sequencing on CD19 + enriched bone marrow samples should be used. As will be discussed in more detail in later sections, *MYD88* and *CXCR4* status are relevant for prognosis and may be relevant to treatment selection. In particular, the minority of patients with the *MYD88*^WT^ genotype have a more aggressive disease than patients with mutated *MYD88* and respond poorly to ibrutinib monotherapy [20].

## BTKi efficacy

The targeting of BTK in B-cell malignancies by ibrutinib has met the expectations engendered by preclinical data and led to the development of several next-generation BTKis. Here, we review the clinical trial data reported for current and emerging BTKis and look beyond the summary efficacy data to understand what differentiates them.

### Ibrutinib

The first-generation BTKi ibrutinib was approved in 2015 for the treatment of WM in Europe [23]. The efficacy of ibrutinib monotherapy in previously treated patients with WM was demonstrated in a pivotal phase II trial (NCT01614821) (Table 1), which reported an overall response rate (ORR) and a major response rate (MRR) of 90.5% and 79.4%, respectively, at a median follow-up of 59 months [24, 25]. The 5-year progression-free survival (PFS) rate was 54% for all patients, and overall survival (OS) at 5 years was 87% [24]. This study established ibrutinib as a highly active treatment in pretreated patients, but there were differences in response rates between patients according to their *MYD88* and *CXCR4* mutational status. Almost all *MYD88*^MUT^/*CXCR4*^WT^ patients had a major response to ibrutinib (97.2%) and almost half had a very good partial response (VGPR; 47.2%). Patients with the *MYD88*^MUT^/*CXCR4*^MUT^ genotype had poorer responses (MRR: 68.2%; VGPR: 9.1%), but the poorest responses were reported for the four patients with *MYD88*^WT^/*CXCR4*^WT^ status, none of whom achieved a major response [24].

**Table 1:** BTKi monotherapy outcomes from phase II/III trials.

BTKi	Publication	Description (phase, patients)	Outcomes	Safety
Ibrutinib	Treon et al. [24, 25]	Phase II, R/R (*N* = 63)	Median FU 59 months: ORR: 90.5% MRR: 79.4% PFS: 2 years, 69.1% OS: 2 years, 95.2% OS: 5 years, 87% Median time to response: 4 weeks	59 months median FU: Grade ≥3 AEs: neutropenia (15.9%), thrombocytopenia (11.1%), and pneumonia (3.2%). AF: all grades 12.7%.
	Dimopoulos et al. [29]	Phase III, rituximab-refractory (*N* = 31)	Median FU 18.1 months: ORR: 90% MRR: 71% PFS: 18 months, 86% OS: 18 months, 97%	Common Grade ≥3 AEs: neutropenia (13%); hypertension (10%); and anemia, thrombocytopenia, and diarrhea (all 6%).
	Castillo et al. [30]	Phase II, TN (*N* = 30)	Median FU 50 months: ORR: 100% MRR: 87% VGPR: 30% PFS: 4-year, 76% OS: 4-year, 100%	Grade 2–4 AEs: Fatigue (33%); upper respiratory tract infection (30%); hematoma (27%); atrial fibrillation and urinary tract infection (both 20%); hypertension, lower tract respiratory infection, and rash (all 17%)
Zanubrutinib	Trotman et al. [95]	Phase I/II, TN (*n* = 24) and R/R (*n* = 53)	Median FU 30 months: ORR: 95.9% MRR: 82.2% VGPR + CR: 45.2% PFS: 3-year, 81% OS: 3-year, 85%	The most common all-grade AEs: upper respiratory tract infection (51.9%), contusion (32.5%), and cough (22.1%). Grade ≥3 AEs: neutropenia 15.6%; anemia 9.1%; and basal cell carcinoma and cellulitis (both 5.2%).
Zanubrutinib versus ibrutinib	Tam et al. [32], ASPEN trial	Phase III, R/R (*n* = 164) and TN (*n* = 37)	Median FU 19.4 months: Zanubrutinib ORR: 94% MRR: 77% VGPR: 28% PFS: 18 months, 85% OS: 18 months, 97% Ibrutinib ORR: 93% MRR: 78% VGPR: 19% PFS: 18 months, 84% OS: 18 months, 93%	AEs (zanubrutinib versus ibrutinib): All-grade neutropenia: 13% vs 19% Pneumonia: 2% vs 12% AF: 2% vs 15%
Zanubrutinib	Dimopoulos et al. [33], ASPEN trial extension	Phase III, *MYD88*^WT^ (23 R/R and 5 TN)	Median FU 17.9 months: ORR: 81% MRR: 50% VGPR: 27%	AEs similar to those reported by Tam et al [32] in the head-to-head arm of the ASPEN trial
Acalabrutinib	Owen et al. [34]	Phase II, R/R (*n* = 92) and TN (*n* = 14)	Median FU 27.4 months: R/R ORR: 93% TN ORR: 93%	Grade 3–4 AEs >5%: neutropenia (16%) and pneumonia (7%). Grade 3–4 AF: 1%. Grade 3–4 bleeding: 3%.
Tirabrutinib	Sekiguchi et al. [35]	Phase II, TN (*n* = 19) and R/R (*n* = 9)	Median FU 6.5–8.3 months: MRR: 88.9% ORR: 96.3%	Common all-grade AEs: rash (44.4%), neutropenia (25.9%), and leukopenia (22.2%). Grade ≥3 AEs: neutropenia (11.1%), lymphopenia (11.1%), and leukopenia (7.4%). All bleeding events were Grade 1; no AF or hypertension.
Pirtobrutinib	Mato et al. [37]	Phase I/II, R/R B-cell malignancies (*N* = 323); WM (*n* = 19)	In WM patients (*n* = 19): ORR: 68% MRR: 47%	In all patients (*N* = 323): Grade ≥3 neutropenia: 10%; all grade AF: 1%.

*AF* atrial fibrillation, *FU* follow-up, *MRR* major response rate, *ORR* overall response rate, *PFS* progression-free survival, *R/R* relapsed/refractory, *TN* treatment-naïve, *VGPR* very good partial response.

The phase III iNNOVATE trial (NCT02165397) compared the combination of ibrutinib plus the anti-CD20 monoclonal rituximab versus rituximab monotherapy in patients with WM. The combination resulted in significantly higher response rates compared with rituximab monotherapy, regardless of genotype. Notably, at a median follow-up of 50 months, patients with the *MYD88*^WT^/*CXCR4*^WT^ genotype achieved robust responses with ibrutinib plus rituximab, with an ORR of 82% and an MRR of 73%, including 27% VGPRs. An ORR of 100% was reported for patients with the *MYD88*^L265P^/*CXCR4*^WHIM^ genotype, which included 77% MRRs and 23% VGPRs. The results suggest that ibrutinib plus rituximab may benefit patients with the *MYD88*^WT^ genotype who respond poorly to ibrutinib monotherapy [26, 27]. It is important to state that the iNNOVATE trial did not include an ibrutinib monotherapy arm and only small numbers of patients were represented for some genotypes, such as *MYD88*^WT^ [26, 27]. However, particularly for patients with *CXCR4* mutations, ibrutinib plus rituximab is an attractive treatment that may overcome delayed and suboptimal treatment responses that have been reported with ibrutinib monotherapy [24].

ICT regimens based on rituximab are the mainstay of first-line treatment in WM, so it is essential to assess the effectiveness of ibrutinib post rituximab failure [28]. A substudy of the iNNOVATE trial in rituximab-refractory patients showed that ibrutinib monotherapy was associated with an MRR of 71%, a PFS rate of 86%, and an estimated 18-month OS rate of 97% at a median follow-up of 18.1 months [29]. These outcomes were notable given that the substudy population had a median of four previous lines of therapy [29].

Ibrutinib’s activity in the first-line setting was demonstrated in an open-label trial (NCT02604511) of 30 treatment-naïve (TN) patients with WM, all of whom had the *MYD88*^L265P^ mutation [30]. At 50 months of follow-up, MRR and VGPR rates were 87% and 30%, respectively, and median PFS was not reached. VGPR rates were numerically, but not significantly, lower for patients with *CXCR4* mutations (14% vs 44%; *P* = 0.09). The 4-year PFS rates reflected this trend (59% vs 92%; *P* = 0.06) [30].

### Zanubrutinib

The covalent BTKi zanubrutinib was approved by the European Medicines Agency in November 2021 for the treatment of adult patients with WM who have received at least one prior therapy, or in first-line treatment for patients unsuitable for ICT [31]. In the phase III ASPEN trial (NCT03053440) comparing zanubrutinib with ibrutinib in *MYD88*^L265P^ patients, rates of overall and deep responses were comparable between both molecules (Table 1) [32]. There were no significant differences for zanubrutinib versus ibrutinib in ORR (94% vs 93%), MRR (77% vs 78%), PFS (85% vs 84%), or OS (97% vs 93%) at 18 months [32]. The trial did not meet its primary endpoint of more complete responses (CRs) or VGPRs with zanubrutinib versus ibrutinib. However, there was a trend toward deeper responses in favor of zanubrutinib (VGPR: 28% vs 19%; *P* = 0.09) [32].

Median time to a major response for both arms was 2.8 months, and median time to VGPR was very similar for zanubrutinib versus ibrutinib in R/R patients (4.7 vs 5.1 months) [32]. Interestingly, TN patients treated with zanubrutinib had a much faster median time to VGPR than the ibrutinib-treated patients (5.6 vs 22.1 months). TN patient numbers were low (19 zanubrutinib and 18 ibrutinib) [32], but these data suggest a possible advantage for the use of zanubrutinib compared with ibrutinib in the first-line setting.

The proportion of patients in the ASPEN study with the *CXCR4*^WHIM^ mutation detected by Sanger sequencing in the primary analysis was significantly lower (9%) than expected. A post hoc analysis employing next-generation sequencing of bone marrow samples from 95% of patients detected *CXCR4*^WHIM^ in 28% of all patients, which comprised 34% of patients in the zanubrutinib arm and 22% in the ibrutinib arm. The VGPR rate for zanubrutinib-treated patients with *CXCR4*^WHIM^ was lower than for patients with *CXCR4*^WT^ (29% vs 34%). Zanubrutinib-treated patients with *CXCR4*^WHIM^ had a higher VGPR rate than those treated with ibrutinib (29% vs 21%) [32].

Given the poor responses reported for ibrutinib in patients with the *MYD88*^WT^ genotype, all 26 patients with *MYD88*^WT^ status were assigned to a separate arm of the ASPEN trial to receive open-label zanubrutinib. Importantly, the study employed a two-step process to detect *MYD88* mutations with a PCR-based method for detection of L265P followed by next-generation Sanger sequencing of *MYD88* in patients negative for L265P. Compared with previously reported outcomes with ibrutinib, *MYD88*^WT^ patients responded well to zanubrutinib, with 50% of patients achieving a major response and 27% a VGPR at 18 months [33]. Only cautious conclusions can be made based on cross-trial comparisons and the molecular basis of any disparity in the responses of this patient subset to zanubrutinib versus ibrutinib is unclear. However, the ASPEN study authors noted that zanubrutinib achieves full versus partial occupancy of BTK in blood and lymph nodes over 24 hours versus ibrutinib [33]. This may provide a greater potential for deep and sustained remissions in patients with more aggressive disease.

To date, the ASPEN study is the only clinical trial to directly compare two different BTKis in WM. It demonstrated the high efficacy and safety of zanubrutinib and ibrutinib in the treatment of WM. However, no patients in either arm of the trial achieved a CR [32, 33], which highlights the fact that eradication of WM remains a challenge with BTKi monotherapy.

### Acalabrutinib

Acalabrutinib is another covalent BTKi that has undergone clinical assessment in WM, but it is not currently approved for the treatment of WM in Europe. A single-arm, phase II study (NCT02180724) of acalabrutinib reported a 24-month PFS rate of 90% for TN patients and 82% for R/R patients. At a median duration of follow-up of 27.4 months, ORRs were 93% for TN and R/R patients [34]. VGPRs were reported for 9% of R/R patients and no TN patients, and there were no CRs. The limitations of cross-trial comparisons apply, but the VGPR rates reported for acalabrutinib are lower than the rates reported in the ASPEN study for zanubrutinib (TN: 26%; R/R: 29%) or ibrutinib (TN: 17%; R/R: 20%) after a similar follow-up period (median: 19.4 months) [32].

Patients with *MYD88*^WT^ status treated with acalabrutinib appear to have poorer outcomes than *MYD88*^MUT^ patients, with ORRs of 79% vs 94%, respectively. No VGPRs or CRs were reported for these patients [34].

### Other next-generation BTKis

Data for other BTKis in WM are very limited (see Table 1). Outcomes from a small, multicenter, phase II study (JapicCTI-173646) of the covalent BTKi tirabrutinib in TN and R/R WM patients (MRR: 88.9%; ORR: 96.3%) were similar to outcomes with other BTKis. However, the study numbers were too small to draw conclusions about the impact of genotype [35].

Non-covalent, reversible inhibitors of BTK have the potential for greater target specificity than covalent BTKis and could be a valuable option for patients resistant to covalent BTKis [36]. Pirtobrutinib (formerly known as LOXO-305) was reported to have good efficacy in the phase I/II BRUIN study (NCT03740529) in patients with R/R B-cell malignancies. An ORR of 68% was reported for 19 patients with R/R WM, with partial responses in 50% of patients (no CRs or VGPRs). In 13 patients who had previously received a covalent BTKi, the ORR was 69%, with partial responses in 38% of patients [37].

## Ibrutinib tolerability and patient management

Ibrutinib is associated with a range of side effects that are usually manageable but do result in significant rates of discontinuations. In a retrospective analysis of real-world outcomes for 80 patients with WM, 17 patients (21%) had discontinued ibrutinib because of treatment-related toxicities at a median follow-up of 19 months [38]. AF and elevated liver enzymes (*n* = 3 patients for both; 18%) were the most common side effects leading to discontinuation. Ibrutinib-induced hepatotoxicity is a rare but potentially serious side effect, and close monitoring of liver function is justified; elevated transaminases may be the first sign of hepatotoxicity [39]. Other reasons for discontinuation included uncontrolled infection (2 patients; 12%), fatigue with petechial rash (1 patient; 6%), and blistering rash (1 patient; 6%) [38].

For outcomes with ibrutinib treatment in larger patient populations, it is necessary to consider studies of patients with other B-cell malignancies. A retrospective study from Mato et al. reported outcomes and toxicities for 616 patients with chronic lymphocytic leukemia / small lymphocytic lymphoma (CLL/SLL) in the USA treated with ibrutinib as monotherapy (86%) or within combination regimens (14%) [40]. At a median follow-up of 17 months (range: 1–60 months) 21% of patients had discontinued ibrutinib because of toxicity. In first-line therapy, arthralgia (41.6%), AF (25%), and rash (16.7%) were the leading causes of discontinuation. In subsequent lines of therapy, AF (12.3%), infection (10.7%), pneumonitis (9.9%), bleeding (9%), and diarrhea (6.6%) were the most cited reasons for discontinuation [40]. The rate of discontinuations subsided from the date of ibrutinib initiation, with >75% of total discontinuations occurring within 1 year and >95% within 2 years. Rash was typically the earliest AE to result in discontinuation (median: 3.5 months), followed by pneumonitis (4.5 months), arthralgia (5 months), infection (6 months), atrial fibrillation (7 months), diarrhea (7.5 months), and bleeding (median: 8 months) [40].

Arthralgia/myalgia associated with ibrutinib is usually mild or moderate in severity and most common in the first few months following treatment, although it can occur late in the treatment course [41, 42]. These events are usually tolerable and may resolve without intervention, but in some cases may significantly reduce a patient’s quality of life and become intolerable [41, 42]. Stephens and Byrd recommend that patients with CLL/SLL and intolerable arthralgia are treated with acetaminophen or short pulses of prednisone therapy while continuing ibrutinib at the full dose [42]. Where possible, the use of platelet-inhibiting agents, such as ibuprofen, should be avoided because of the bleeding risk associated with ibrutinib, but they may be introduced if acetaminophen and steroid therapy is not effective. As a last resort, Stephens and Byrd recommend holding ibrutinib for 1 week before restarting at one dose level lower (e.g. 420 mg to 280 mg) [42]. This broadly concurs with the results of Rhodes et al., who found in a retrospective study that dose holds were more effective than dose reductions in patients with CLL/SLL treated with ibrutinib who required management of arthralgia/myalgia [41].

Rash is another common side effect of ibrutinib that is usually low grade. Pruritic rash may be managed with topical corticosteroid therapy and oral antihistamines. In rare cases, dose holds may be considered, but dose reductions and discontinuations are rarely necessary [43]. Diarrhea is another side effect that is frequently reported with ibrutinib, but is rarely severe and usually resolves without intervention [42].

Cytopenias are another common side effect of ibrutinib. Ibrutinib-associated neutropenia is common in WM treatment and may be associated with an increased risk of infections. Neutropenia associated with ibrutinib treatment is more easily reversed than some ICT-associated neutropenia in WM. Fludarabine, for example, causes prolonged and potentially irreversible cytopenias that are associated with a significantly increased risk of infection [44, 45]. In the case of neutropenia caused by ibrutinib, granulocyte colony-stimulating factor support may be used to rescue Grade ≥3 neutropenia. Ibrutinib dose reductions are not usually necessary but may be considered for recurrent neutropenia.

Conversely, ibrutinib may cause B cell lymphocytosis upon initiation, which is usually transient but may be prolonged in some patients. This is thought to be a class effect caused by disruption to B-cell homing mechanisms, which results in distribution of malignant B cells into the peripheral blood. Although lymphocytosis is usually asymptomatic and does not require management [43], it is important to not mistake lymphocytosis with disease progression in patients treated with ibrutinib or other BTKis.

## Ibrutinib and cardiovascular toxicity

Perhaps the most challenging aspect of treating patients with ibrutinib is the associated cardiovascular adverse drug reactions, of which AF is the most common. Mining the international pharmacovigilance database VigiBase, Salem et al. found that supraventricular arrhythmias (SVAs), of which 93.8% were AF, were 23.1 times overreported with ibrutinib compared with all other drugs within VigiBase [13]. AF rates are usually reported at 5–10% for patients treated with ibrutinib in clinical trials [46], but the true incidence is likely to be higher. With systematic cardiological screening, Baptiste et al. reported an AF rate of 38% at 24 months in patients with B-cell malignancies treated with ibrutinib [47].

**Fig. 3:**
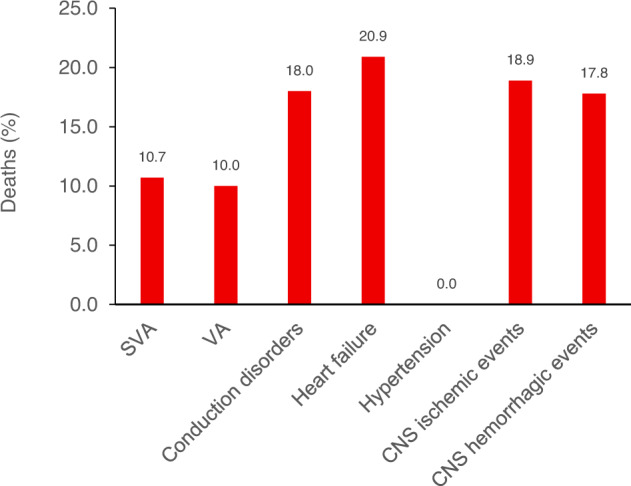
Death in patients with cardiovascular adverse-drug reactions with ibrutinib reported to VigiBase from its creation in November 1967 to February 2018. Adapted from [13]. CNS central nervous system, SVA supraventricular arrhythmia, VA ventricular arrhythmia.

AF is usually manageable, but it is likely to require lifelong management [48] and is associated with an increased risk of mortality (Fig. 3) [13]. A major risk associated with AF is stroke [49] and patients with AF who are treated with ibrutinib should be evaluated for the need for anticoagulants to reduce this risk [50], potentially using the CHA_2_DS_2_-VASc assessment [51]. There is no consensus on the preferred oral anticoagulant for patients who have AF being treated with ibrutinib, although warfarin or other vitamin K antagonists were associated with significant bleeding rates when used in combination with ibrutinib in seminal clinical trials [52, 53]. Low-molecular-weight heparin or factor Xa inhibitors may be considered.

Tachycardia leading to heart failure is the other major risk associated with ibrutinib-associated AF. Lenient rate control with beta-blockers is favored over rhythm control [13] because continuing treatment with ibrutinib may limit a patient’s ability to maintain sinus rhythm after cardioversion [46]. Electrical or chemical cardioversion should be considered only in patients who are still symptomatic after rate control and who can tolerate anticoagulation, because there is an increased risk of thromboembolic events after cardioversion [46].

Ventricular arrhythmia (VA) is a related potential complication of ibrutinib therapy that is less common than AF but was reported to be 4.7 times more likely with the use of ibrutinib compared with all other drugs within VigiBase [13]. VA is the most common cause of sudden cardiac death [54], and the benefits of ibrutinib therapy should be carefully weighed against the risk of worsening VA in patients who have a history of premature ventricular contractions and hyperexcitability.

A risk of hypertension should also be considered and requires long-term vigilance. Unlike AF and VA, which usually occur in the first few months after treatment initiation, hypertension is associated with an increasing risk over time. The RESONATE study followed patients with R/R CLL/SLL treated with ibrutinib for up to 6 years and reported a prevalence of Grade ≥3 hypertension of 4% in years 0–1 and 11% in years 5–6. With a median treatment duration of 41 months, any grade hypertension occurred in 21% of patients [55]. The real-world prevalence of hypertension in patients treated with ibrutinib may be significantly higher than this figure. A retrospective study that followed 562 lymphoma patients treated with ibrutinib over a 17-year period in a real-world setting reported that 71.6% experienced new hypertension [56].

## Ibrutinib and bleeding risk

Bleeding events are common in patients treated with ibrutinib, with most being low grade and occurring within 6 months of initiation [57]. Ibrutinib-associated bleeding may exacerbate disease-specific risk factors for patients with WM. A significant proportion of patients are thrombocytopenic and/or anemic [58] and hyperviscosity caused by high levels of circulating IgM can lead to tearing of small blood vessels, particularly in the nose, gums, or retina [59]. Patients with WM may also develop acquired von Willebrand disease and this risk also appears to be higher in patients with high serum IgM levels [60].

Patients treated with ibrutinib may also require anticoagulants to decrease the risk of stroke associated with AF, and these patients should be monitored particularly carefully, especially in the first few months after ibrutinib initiation. The benefits of other medications associated with an increased bleeding risk, such as aspirin and nonsteroidal anti-inflammatory drugs, should be weighed against the risks. In the case of aspirin, it is recommended that aspirin is stopped for patients with a low or moderate cardiovascular risk, and that those at high risk continue with a dose of ≤81 mg/day [57]. Fish oil and vitamin E supplements are also associated with severe bleeding events and should be avoided [57].

Because of the bleeding risk, it is recommended that ibrutinib is paused perioperatively [57]. The anti-platelet effects of ibrutinib appear to be reversed within a week of discontinuation [61, 62], so pausing ibrutinib for one week prior to, and up to 2–3 days after, surgery is reasonable. Platelet transfusion can reduce the risk of bleeding during unplanned surgeries [57, 61]. The elapsed time since the last dose of ibrutinib should be considered because this will affect the efficacy of a transfusion. Ibrutinib has a half-life of 4–13 hours [23], and transfusions given very soon after the last ibrutinib dose are likely to be less effective in reversing the hemostatic defect.

## Ibrutinib withdrawal

Planned surgery is the most common reason to pause ibrutinib, but it may also be paused because of toxicities, drug interactions, and patient decision [63]. A retrospective single-center study reported ibrutinib-withdrawal symptoms in 19% of patients with WM undergoing ibrutinib pause, with fever, body aches, night sweats, and arthralgias being the most common. One-third of these patients had symptoms associated with progressive disease and two-thirds had symptoms in the absence of progressive disease [63]. Responses are usually regained quickly following ibrutinib resumption [64], but interruptions may be associated with a shorter PFS and should be minimized where possible [63]. Patients experiencing withdrawal may benefit from a short course of prednisone (10 mg twice daily) during the interruption period [63].

## Zanubrutinib tolerability and patient management

**Fig. 4:**
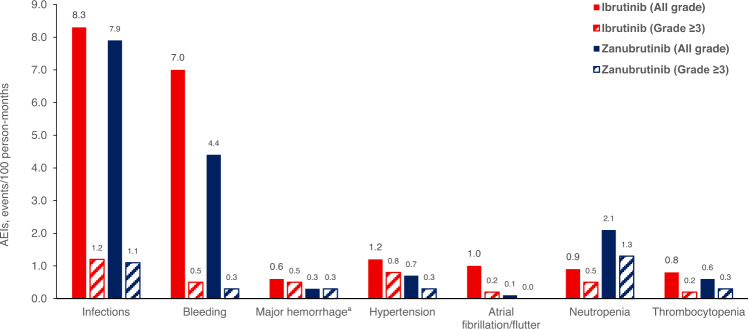
BTK inhibitor class adverse event rates in the ASPEN Phase III trial. All grade and Grade 3 events of interest reported with ibrutinib and zanubrutinib monotherapy in the ASPEN Phase III trial. Adapted from [32]. ^a^Major hemorrhage was defined as serious or grade ≥3 bleeding at any site, or central nervous system bleeding of any grade. AESI adverse event of special interest.

In the phase III, head-to-head ASPEN trial, fewer patients treated with zanubrutinib versus ibrutinib required dose reductions (14% vs 29%) and treatment discontinuation (4% vs 9%). The most common AEs with zanubrutinib were neutropenia (all grades: 29%; Grade ≥3: 20%), upper respiratory infection (all grades: 24%; Grade ≥3: 0%), and diarrhea (all grades: 21%; Grade ≥3: 3%) [32]. The rate of neutropenia for zanubrutinib (all grades: 29%; Grade ≥3: 20%) was more than twice the rate for ibrutinib (all grades: 13%; Grade ≥3: 8%). Febrile neutropenia was also reported in the zanubrutinib arm (all grades: 4%; Grade ≥3: 4%), but not in the ibrutinib arm. Interestingly, the increased rate of neutropenia did not translate into an increased risk of infections with zanubrutinib. Infection events per 100 person-months were almost identical for zanubrutinib and ibrutinib for all-grade (7.9 vs 8.3) and Grade ≥3 infections (1.1 vs 1.2) (Fig. 4) [32]. Incidence of pneumonia was actually higher among patients treated with ibrutinib (all grades: 12%; Grade ≥3: 7%) than in patients treated with zanubrutinib (all grades: 2%; Grade ≥3: 1%). However, more neutropenic patients received granulocyte colony-stimulating factor support in the zanubrutinib arm than in the ibrutinib arm (47% vs 31%) [32], and this intervention should be considered in patients with Grade ≥3 neutropenia.

Preclinical data suggest that ibrutinib, but not zanubrutinib, induces platelet receptor shedding and is associated with extended bleeding times and increased thrombus formation [32, 65]. In the ASPEN trial, the incidence rates of minor and major hemorrhage favored zanubrutinib compared with ibrutinib; however, the incidence rates were similar between the two molecules [32].

Longer-term follow-up is needed to confirm that a lower incidence rate of cardiovascular adverse drug reactions with zanubrutinib versus ibrutinib is reflected in real-world data. However, the increased selectivity of zanubrutinib appears to result in reduced cardiotoxicity. Rates of AF were reduced with zanubrutinib (all grades: 2%; Grade ≥3: 0%) compared with ibrutinib (all grades: 15%; Grade ≥3: 4%), as was the incidence of hypertension (all grades: 11% vs 16%, respectively; Grade ≥3: 6% vs 11%, respectively) [32].

In common with ibrutinib, most adverse events – including neutropenia, infections, and hemorrhage – occur within the first 6–18 months of exposure to zanubrutinib. A pooled safety analysis of zanubrutinib across six trials in patients with B-cell malignancies revealed second primary malignancies and hypertension as possible exceptions, with both associated with relatively flat event-rate curves [66]. Again, this is consistent with data reported for ibrutinib [56, 67]. AF showed no clear relationship to zanubrutinib exposure, but the total number of events was small [66].

## Acalabrutinib tolerability and patient management

Like zanubrutinib, acalabrutinib appears to have a better safety profile than ibrutinib, with a lower incidence of AF and hypertension [34, 68]. In a phase II trial in WM, the most common Grade ≥3 AEs were neutropenia (16%) and pneumonia (7%) [34]. Grade ≥3 bleeding was rare (3%) [34], and preclinical data suggest that acalabrutinib is associated with a decreased bleeding risk compared with ibrutinib [69].

The most common all-grade AE was headache (39%) [34], and this side effect has also been reported at a high incidence in clinical trials of acalabrutinib in other B-cell malignancies [70]. Headache with acalabrutinib is usually mild or moderate in severity and resolves within the first month of treatment without the need for intervention [71]. Low-grade diarrhea is also common with acalabrutinib, reported at similar rates to headache [70].

A highly relevant property of acalabrutinib compared with the other BTKis is that its absorption is significantly reduced in patients who have taken gastric acid–reducing agents. The summary of product characteristics for acalabrutinib recommends suitable spacing for administration of H2 blockers and antacids and advises against concomitant use of proton pump inhibitors (Table 2) [72].

**Table 2:** Pharmacology and interactions of acalabrutinib, ibrutinib, and zanubrutinib [23, 31, 72].

BTKi	Pharmacokinetics			Metabolism	Interactions
	T_max_ (median)	Volume of distribution at steady state	Half-life (mean)		
Acalabrutinib	0.5–1.5 h	34 L	1–2 h	Primarily CYP3A; to a minor extent by glutathione conjugation and amide hydrolysis	Avoid strong CYP3A4 inhibitors or inducers; gastric acid–reducing agents may decrease the area under the curve of acalabrutinib
Ibrutinib	1–2 h	10 000 L	4–13 h	Primarily CYP3A; to a minor extent by CYP2D6	Avoid strong CYP3A4 inhibitors or inducers
Zanubrutinib	2 h	522 L	2–4 h	Primarily CYP3A	Avoid strong CYP3A inducers; modify the dose of zanubrutinib with moderate or strong CYP3A inhibitors

*CYP* cytochrome P450.

## Tolerability of other BTKis

There are limited safety data for other BTKis. In a trial of 27 patients treated with tirabrutinib, no incidents of AF were reported. Grade ≥3 AEs included neutropenia (11.1%), lymphopenia (11.1%), and leukopenia (7.4%) [35]. The non-covalent, reversible inhibitor pirtobrutinib was reported to have low toxicity in phase I/II trials in patients with R/R B-cell malignancies; notably, there were no incidents of treatment-related AF or hemorrhage [37].

## Tolerability and off-target inhibition

The next-generation BTKis have been developed with the aim of achieving greater tolerability through greater selectivity of BTK. However, the contribution of off-target inhibition to tolerability is not fully understood. Cardiovascular toxicities with ibrutinib are often attributed to off-target inhibition and the greater selectivity of next-generation BTKis potentially translates into a reduced risk of cardiovascular adverse events [73]. Various mechanisms have been proposed for ibrutinib-associated AF, with data from a mouse model indicating that ibrutinib-associated AF may be caused by off-target inhibition of C-terminal SRC kinase [48]. Bleeding with ibrutinib is likely to be caused by a combination of on-target and off-target inhibition as both BTK and TEC have a role in downstream signaling of several platelet transmembrane receptors [57].

## Current role of BTKis in the treatment of WM

Ibrutinib and zanubrutinib are approved for the treatment of R/R WM and for TN patients who are unsuitable for ICT [23, 31]. In addition, ibrutinib in combination with rituximab is approved for all patients with WM regardless of medical fitness. Rituximab-based ICT regimens remain the standard of care in the first-line treatment of patients with WM. These regimens are highly effective, have a manageable toxicity profile, and are fixed-duration regimens that provide the opportunity for treatment-free periods [74]. BTKis offer a high clinical benefit as second- and subsequent lines of therapy, particularly after ICT failure. Therefore, rituximab/chemotherapy and BTK inhibition are pillars for the clinical management of WM today.

## Treatment options following BTKi therapy

BTKis are the treatment standard for patients with R/R WM, but there may be a tendency for some clinicians to reserve BTKis as a therapy of last resort because of the lack of evidence for effective treatments after BTKi failure. Potential options for patients who need to discontinue certain BTKis include other BTKis, proteasome inhibitors, BCL2 inhibitors, PI3Kδ inhibitors, or cellular therapies.

Following the approval of zanubrutinib in November 2021, there is the potential to continue BTK inhibition with zanubrutinib or ibrutinib for patients with WM who discontinue the other BTKi because of toxicities. An ongoing phase II study (NCT04116437) is evaluating the use of zanubrutinib in patients with B-cell malignancies who discontinued ibrutinib or acalabrutinib because of intolerance. At a median follow-up of 4.2 months, zanubrutinib was well tolerated, with no patients needing to discontinue treatment. All efficacy-evaluable patients (*n* = 26) had sustained or improved responses [75].

Non-covalent BTKis, such as pirtobrutinib, have more potential as a salvage therapy for R/R patients with WM previously treated with a covalent BTKi compared with other covalent BTKis. Data from the BRUIN study of pirtobrutinib indicate that non-covalent BTKis are effective in patients who relapse on ibrutinib [37], but there are no data to indicate that the reverse is true. Considering known and probable resistance mechanisms, using non-covalent BTKis after failure of covalent agents may be appropriate in general. Non-covalent inhibitors are active against BTK with the C481S mutation, which is the most common mutation found in ibrutinib-resistant patients [76]. Conversely, mutations to a ‘gatekeeper’ residue in BTK’s ATP pocket, which are predicted to be a probable path of resistance to non-covalent inhibitors [77], also reduce the binding of covalent BTKis [78].

Other options following BTKi failure include proteasome inhibitors and PI3Kδ inhibitors. The proteasome-inhibitor bortezomib is effective as a monotherapy [79] or in combination with rituximab for patients with R/R WM [80]. The BCL2 inhibitor venetoclax is well tolerated and produces high rates of responses in R/R WM, although patients with previous exposure to BTKis may be less likely to have deep responses [81]. Idelalisib, a selective oral inhibitor of PI3Kδ, produces durable responses as a monotherapy in R/R WM [82], as does the combination of idelalisib plus the anti-CD20 monoclonal antibody obinutuzumab [83].

For eligible younger patients, autologous or allogeneic stem cell therapies are options, although these both carry significant risks of non-relapse mortality [84, 85]. Although currently largely confined to clinical trials, chimeric antigen receptor T-cell therapy may in the future be available in the clinic for eligible patients [86, 87]. Indeed, the anti-CD20-targeted autologous CAR T-cell therapy MB-106 has recently been granted orphan drug designation by the FDA. It is currently being investigated in patients with relapsed or refractory CLL and various B-cell non-Hodgkin’s lymphomas, including WM, in a multicenter phase 1/2 trial (NCT05360238) of 287 patients that is scheduled to complete in September 2026.

Bispecific antibodies that engage both B cells and T cells are another emerging therapeutic class with potential across B-cell malignancies. For WM, the CD20/CD3-targeting antibodies mosunetuzumab and glofitamab are the most advanced relevant bispecific antibodies. There are promising data for their activity in other B cell lymphomas, particularly follicular and diffuse large B-cell lymphoma, but expanded trials are required in WM [88].

## Future outlook: combination therapies

Limitations of BTKi monotherapy include the need for indefinite use rather than a fixed duration of treatment and relatively low rates of VGPRs and CRs. Patients with the *MYD88*^WT^ genotype also appear to have poorer outcomes compared with those with the *MYD88*^L265P^ mutation, although data from the ASPEN trial substudy indicate that zanubrutinib may be effective in these patients [33]. To overcome these limitations in the future, many patients may use BTKis as part of combination regimens.

Ibrutinib inhibits some of the anti-tumor activity of anti-CD20 monoclonals through inhibition of antibody-dependent cell-mediated cytotoxicity and phagocytosis [89, 90]. Zanubrutinib and acalabrutinib have less of an inhibitory effect on the activity of anti-CD20 monoclonals [89–91] and may be more suitable for use in combination regimens with anti-CD20 monoclonals. Promising results have been reported for the combination regimens of zanubrutinib plus obinutuzumab in patients with CLL/SLL and follicular lymphoma [92] and acalabrutinib plus obinutuzumab in CLL/SLL [93].

The potential for effective fixed-duration regimens with BTKis has been demonstrated by the multicenter phase II CAPTIVATE study in CLL/SLL of ibrutinib with venetoclax. Primary results from the study suggest the combination offers the potential for treatment-free remission in patients with CLL/SLL with a fixed-duration regimen [94]. There are other active trials in WM investigating the combination of BTKis with various agents, including chemotherapeutics, monoclonal antibodies, proteasome inhibitors, and targeted inhibitors (Table 3).

**Table 3:** Ongoing clinical trials of BTKi combinations for treatment of patients with WM.

Trial	Treatments (class)	Description	Estimated primary completion date
NCT04260217	Ibrutinib and APG-2575 (BCL2 inhibitor)	Phase Ib /II, open label	September 2022
NCT03679624	Ibrutinib and daratumumab (anti-CD38 monoclonal antibody)	Phase II, open label	October 2022
NCT03620903	Ibrutinib and bortezomib (proteasome inhibitor), and rituximab	Phase II, open label	December 2022
NCT04274738	Ibrutinib and mavorixafor (CXCR4 inhibitor)	Phase I, open label	January 2023
NCT03225716	Ibrutinib and ulocuplumab (anti-CXCR4 monoclonal antibody)	Phase I/II, open label	January 2023
NCT04463953	Zanubrutinib, ixazomib (proteasome inhibitor), and dexamethasone	Phase II, open label	May 2023
NCT04273139	Ibrutinib and venetoclax (BCL2 inhibitor)	Phase II, open label	June 2023
NCT04624906	Acalabrutinib, bendamustine, and rituximab	Phase II, open label	December 2024
NCT03506373	Ibrutinib and ixazomib (proteasome inhibitor)	Phase II, open label	May 2025
NCT04263480	Ibrutinib and carfilzomib (proteasome inhibitor)	Phase II, open label	February 2028

## Conclusion

Clinical trial data suggest there are only limited differences in efficacy against WM between the different BTKis, although zanubrutinib and acalabrutinib may produce faster and deeper responses for patients with the mutated *CXCR4* and *MYD88*^WT^ genotype compared with ibrutinib. However, data from these same studies indicate that zanubrutinib and acalabrutinib have improved safety profiles in comparison with ibrutinib, including a reduction in the risk of cardiovascular side effects. Currently, the options for anti-BTK treatment of WM in Europe are ibrutinib and zanubrutinib.

BTKis are established as effective and well-tolerated treatment options for patients with WM, and next-generation BTKis provide additional advantages that strengthen the role of this class in lymphoma treatment. A remaining key challenge with BTKi therapy is the need for continuous treatment versus fixed-duration and treatment-free intervals afforded by ICT. The future of BTKi treatment in WM and other B-cell malignancies is likely to be as part of combination regimens that offer deep responses with a fixed duration of treatment.

## Data Availability

Data sharing is not applicable to this article as no datasets were generated or analysed during the current study.
